# Sodium nitroprusside improves circulatory failure in rabbit acute pulmonary embolism combined with shock model possibly by enhancing NO release and inhibiting TLR4/NF-кB/HIF-1α signaling pathway

**DOI:** 10.3389/fphys.2025.1573405

**Published:** 2025-07-01

**Authors:** Yuchan Zhou, Yuting Wang, Delong Yu, Xiaoyan Liu, Wenjuan Cao, Huanhuan Li, Ye Gu, Liqun Hu, Yupeng Bai

**Affiliations:** ^1^ Department of Cardiology, Wuhan Fourth Hospital, Puai Hospital, Wuhan, China; ^2^ Hubei University of Medicine, Shiyan, China

**Keywords:** acute pulmonary embolism, sodium nitroprusside, TLR4, NF-κB, HIF-1α

## Abstract

Sodium nitroprusside (SNP) is a fast-acting non-endothelium-dependent vasodilator, which is commonly used to treat diseases such as hypertensive emergencies and acute heart failure. However, there are few experimental studies evaluating the effects and mechanism of SNP on acute pulmonary embolism. To explore the mechanism of action of SNP in acute pulmonary embolism combined with shock, we randomly divided rabbits into sham operation group (S group, n = 8), model group (M group, n = 8), and SNP group (SNP group, n = 8). The rabbit model of acute massive pulmonary embolism with circulatory shock was established by injecting autologous blood clots into the pulmonary artery. Pulmonary angiography showed that the mean pulmonary artery pressure increased significantly and the mean arterial pressure decreased significantly in the model group, which could be reversed by SNP treatment. In addition, the TLR4/NF-κB/HIF-1α pathway signaling pathway was activated in the embolic and non-embolic areas of the lung tissue in the M group, promoting the release of inflammatory factors such as IL-6 and TNF-α, downregulation of endogenous NO expression in the model group. After SNP treatment, the expression of TLR4/NF-κB/HIF-1α pathway signals in the embolic and non-embolic areas was downregulated, the release of inflammatory factors was reduced, the release of vascular NO was increased. Our study shows that SNP has a protective effect on acute pulmonary embolism combined with shock. Its protective effect may be related to inhibiting the TLR4/NF-κB/HIF-1α signaling pathway and increasing NO release in the embolic and non-embolic areas, thereby dilating pulmonary blood vessels, reducing the release of inflammatory factors and partially reversing circulatory failure in this model.

## Introduction

Despite diagnostic and treatment advances, mortality of acute pulmonary embolism (APE) remains high within 3 months of disease onset (Becattini and Agnelli, 2008). Deepened understanding of disease pathogenesis is of importance to improve prognosis of patients with APE. Previous studies have shown that inflammatory response plays a crucial role on aggravating lung injury post APE (Kulkarni et al., 2022). We previously showed that pulmonary vasospasm in the embolic area and non-embolic area jointly led to pulmonary blood flow attenuation in rabbit model of APE combined with shock (Yu et al., 2018). However, the detailed mechanism of pulmonary vasospasm in the lung tissue and non-pulmonary tissue of APE is not fully understood. Previous studies have shown that sodium nitroprusside (SNP) treatment can improve systemic blood flow and increase MAP (mean arterial pressure) (Arnaud et al., 2012), and the formation of hypoxic pulmonary hypertension is related to the reduction of endothelial Nitric oxide (NO) caused by the NO-cGMP pathway (Christou et al., 2018). Our previous study demonstrated that post SNP therapy, NO release was increased dramatically and the inflammatory response in the lung tissue was alleviated, leading to attenuated inflammation-mediated damage, as well as restored pulmonary blood flow in the rabbit APE model with shock (Wang et al., 2020). Base on previous research findings, we hypothesize that the administration of sodium nitroprusside induces a substantial release of NO in both embolized and non-embolized regions, thereby alleviating pulmonary vascular spasm across the entire lung tissue. This mechanism indirectly suppresses the signaling pathway, consequently mitigating downstream inflammatory damage. To test this hypothesis, we explored the changes in Toll-like recetor 4 (TLR4), phosphorylated nuclear factor κB (p-NF-κB), Nuclear Factor κB (NF-κB), Hypoxia inducible factor-1α (HIF-1α), Galectin-3 (Gal-3), Interleukin-6 (IL-6), Tumor necrosis factor-α (TNF-α) and NO in the embolic and non-embolic areas of the lung tissue in the rabbit APE combined with shock model.

## Methods

### Animals

All animal experiments followed the Guide for the Care and Use of Laboratory Animals of the National Institutes of Health (NIH Publication No. 85–23, revised in 1996), and the study protocol was approved by the Animal Care Committee of Huazhong University of Science and Technology (NO. 20160298). Healthy adult New Zealand rabbits (weight 2.5–3.0 kg) were purchased from the Experimental Animal Center of Tongji Medical College, Huazhong University of Science and Technology. All rabbits were housed under standard conditions for 1 week before the experiment and had free access to food and drinking water. All rabbits were fasted from food and water for 12 h before surgery. All surgeries were performed under sodium pentobarbital [3% sodium pentobarbital (20 mg/kg)] anesthesia through the marginal ear vein. After anesthesia, the rabbits were fixed on the operating table in a supine position, and the heart rate and blood pressure were monitored in real time using a LEAD-7000 monitor (Sichuan Jinjiang Electronic Technology Co., Ltd., China).

### Model establishment

First, the skin of the right inguinal area was prepared and disinfected, and the femoral artery and vein were separated and punctured using the Seldinger puncture method. Under X-ray fluoroscopy, a 4F Cordis catheter (Cordis Corporation, Florida, United States) was placed into the main pulmonary artery through the femoral vein. The 4F Cordis catheter was connected to a pressure sensor, and the mean arterial pressure (MAP) and mean pulmonary artery pressure (MPAP) were monitored in real time using a LEAD-7000 monitor (Sichuan Jinjiang Electronic Technology Co., Ltd., China). A 5F sheath (Radiofocus TERUMO) was inserted into the femoral artery. Second, an autologous blood clot [3 mm (±0.5) × 10 mm] was prepared and placed at the end of the 4F catheter, and 5 mL of normal saline was slowly injected into the rabbit pulmonary artery. The autologous blood clot was delivered to the main pulmonary artery in two stages. In the first stage, one blood clot was injected four times at intervals of 1 min. Second, if the intial model fails to meet the success criteria, the blood blot will be readministrated at 60-s intervials during the second phase, and all rabbits reached the shock status within a maximal additional three clot injections in this experiment. Finally, Model success criteria were as follows: (a) MAP decreased to <60 mmHg within 2 min after final clot injection; (b) MPAP increased to 2 times of the baseline level within 2 min after final clot injection. All rabbits in our experiment reached the state of shock.

### Grouping and drug administration

Twenty-four rabbits were divided into sham operation group (S group, surgery was performed as model establishment described above, normal saline instead of clot was injected, n = 8), APE combined with shock model (M group, n = 8), SNP group (SNP group, n = 8, SNP was infused into the pulmonary artery, 3.5 μg/kg/min for 120 min).

### Hemodynamic monitoring

Heart rate were continuously monitored using the LEAD-7000 monitor from Sichuan Jinjiang Electronic Technology Co., Ltd., China. MPAP and MAP were recorded before embolization, after successful modeling, and 120 min after successful modeling. Blood gas analysis was performed at above time points. One ml of blood was collected through the femoral artery sheath using an arterial cannula (BD Biosciences, San Jose, CA), PO_2_ values were determined by a blood gas analyzer (GEM Premier 3000; Instrument Laboratory (Orangeburg, NY).

### Pulmonary angiography

Pulmonary artery angiography images were obtained through pulmonary artery angiography (AlluraXper FD20 Philips).

### Pathological examination

At the end of the experiment, the rabbits were killed under deep anesthesia in that 3% sodium pentobarbital (20 mg/kg) was injected through the ear vein. Tissues from 3 rabbits in the S group, 3 rabbits in the M group, and 3 rabbits in the SNP group were randomly taken for pathological examination including hematoxylin-eosin (HE) and immunohistochemical staining. Briefly, lung tissues were removed and fixed with paraformaldehyde. After paraffin embedding, tissue from PE (pulmonary enbolism) area and non-PE (non-pulmonary enbolism) area was sectioned for pathological examination. Lung tissue sections prepared for immunohistochemical staining were immunohistochemically stained for Gal-3 (1: 300), TLR4 (1: 200), NF - κB (1: 200). Pathological changes were quantified using commercial softwares (Image-Pro Plus, Media Cybernetics, Inc., Rockville, Maryland, United States) and evaluated by evaluating the percentage of positive areas.

### Biochemical analysis

The levels of NO (Nanjing Jiancheng Bioengineering Institute, Nanjing, China) in the embolized and non-embolized areas were measured by ELISA.

### Real-time polymerase chain reaction assay

RNA was extracted from lung tissues in the embolic and non-embolic areas using RNA Tissue Mini Kit (Takara, Dalian, Liaoning, China). Reverse transcription and cDNA synthesis were performed using PrimeScript RT Master Mix (Perfect Real Time, Takara, Dalian, Liaoning, China). Real-time polymerase chain reaction was used to detect the mRNA expression of various cytokines, including Toll-like receptor 4 (TLR4), hypoxia-inducible factor-1α (HIF-1α), galectin-3 (Gal-3), interleukin-6 (IL-6), and tumor necrosis factor α (TNF-α). Specific running procedures: Predegeneration: 95.0°C: 30s; Denature: 95.0°C:15s; Anneling: 55.0°C:15s; Extension: 72.0°C: 30s; Cycle: 40. The primer sequences are shown in Supplementary Table S3.

### Western blotting

Lung tissues were lysed in frozen RIPA buffer containing protease inhibitors, and total lung tissue proteins were extracted from PE and non-PE areas, respectively. Protein concentrations were determined by BCA method, separated by SDS-polyacrylamide gel electrophoresis, and then transferred to polyvinylidene fluoride (PVDF) membranes. PVDF membranes were blocked with 5% skim milk powder dissolved in tris-buffered saline containing Tween 20 (TBST) buffer at room temperature for 2 h, and incubated with HIF-1α (1:1000), Gal-3 (1: 2000), TLR4 (1: 1000), p-NFκB (1:1000), NFκB (1:1000), and IL-6 (1:1000) overnight at 4°C (Supplementary Tables S1, S2). After washing three times with TBST, the cells were incubated with secondary antibodies, and the protein band map was obtained by chemiluminescence for semi-quantitative analysis (Bio-Rad, United States).

### Statistical analysis

All statistical data are expressed as mean ± SD. All data were first analyzed by Shapiro-Wilk test. Normal distributed data were analyzed by one-way analysis of variance with Tukey’s *post hoc* test for the differences between multiple groups. Data that did not conform to normal distribution were evaluated for differences between groups using non-parametric tests. Analysis was performed using IBM SPSS 22.0 software. P < 0.05 was considered statistically significant.

## Results

### Hemodynamics

**FIGURE 1:**
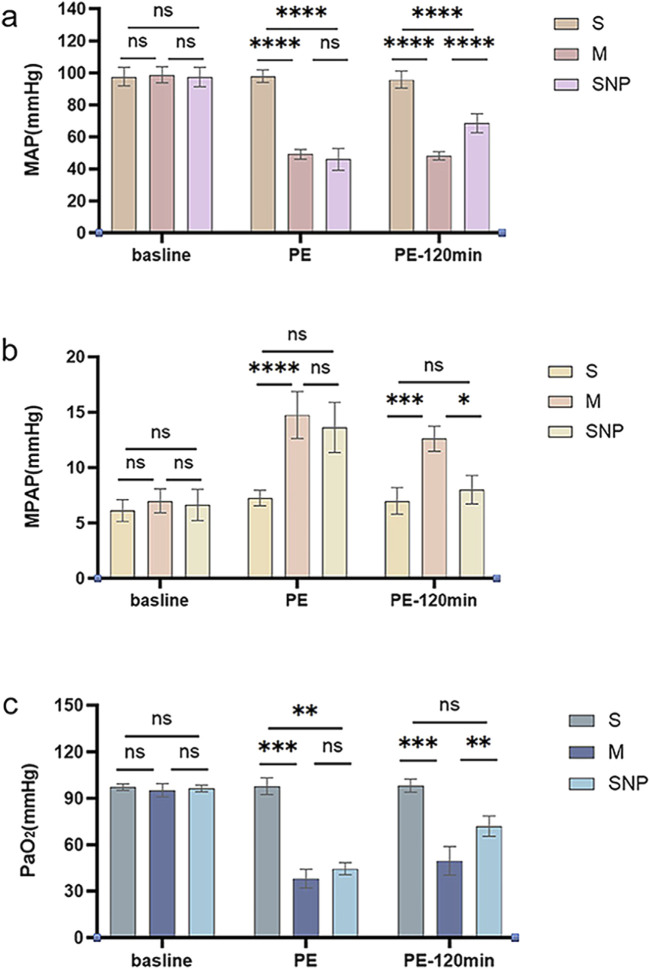
Pressure Monitoring. **(A)** mean arterial pressure (MAP) changes and **(B)** mean pulmonary artery pressure (MPAP) and PaO_2_ analysis **(C)** among various groups. ns *P >* 0.05, *P < 0.05, **P ≤ 0.01; ***P ≤ 0.001, ****P ≤ 0.0001. S, sham operation group; M, model group; SNP, sodium nitroprusside group. Baseline, before pulmonary embolism; PE, pulmonary embolism referring to the moment of successful modeling; PE-120min, at 120 min after successful modeling.

MPAP and MAP levels of each group were similar at baseline. Compared with the S group, the MAP of the M and SNP groups was significantly reduced at the moment of successful modeling (*P* < 0.05, Figure 1a). The MAP value of the SNP group was significantly higher than that of the M group at 120 min after successful modeling (*P* < 0.05, Figure 1a). The MPAP value of the M and SNP groups were significantly higher compared with the S group at the moment of successful modeling (P < 0.05, Figure 1b). At 120 min after successful modeling, the MPAP of the SNP group was significantly lower than that of the M group (P < 0.05, Figure 1b).

### Blood gas analysis

PaO_2_ was measured before embolization, at the moment of successful modeling, and at 120 min after successful modeling. The baseline PaO_2_ levels were similar among groups. After successful modeling, the PaO_2_ of the M and SNP groups were significantly lower than that of the S group (P < 0.05, Figure 1c); at 120 min after the successful modeling, PaO_2_ of the SNP group was significantly higher than that of the M group (P < 0.05, Figure 1c).

### Pulmonary angiography

**FIGURE 2:**
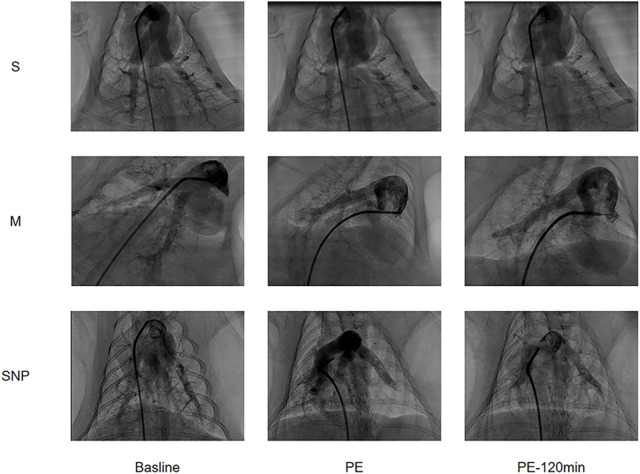
Pulmonary Angiography images arranged in a 3x3 grid showing three different labels (S, M, SNP) across three time points (Baseline, PE, PE-120 min). Each row represents a different condition (S, M, SNP) and each column shows the progression over time. The images display contrast-filled blood vessels or structures, with visible changes of Pulmonary Embolism.

Pulmonary angiography images were obtained at baseline, at the moment of successful modeling, and at 120 min after the successful modelling (Figure 2). The blood flow conditions in each group were similar at baseline, reduced in M and SNP groups and was significantly improved at 120 min in SNP group as compared to M group.

### HE pathological results

**FIGURE 3:**
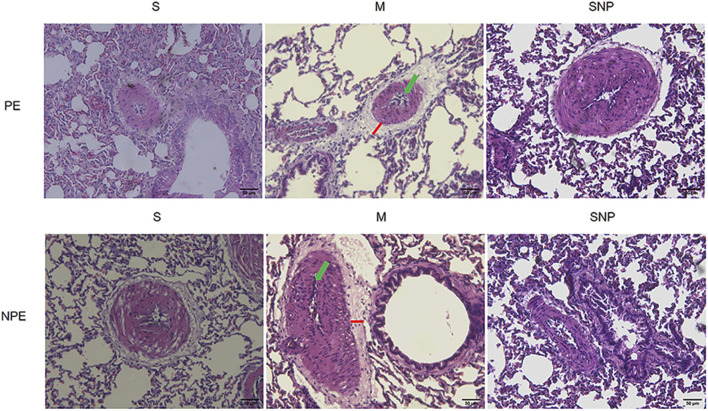
Histological features of pulmonary embolism (PE) and non-pulmonary embolism (NPE) tissues on hematoxylin and eosin (HE) stained sections. Red lines indicate the interstitial edema and green arrows represents the inflammatory cell infiltration. PE, pulmonary embolism tissue; NPE, non-pulmonary embolism tissue. S, sham operation group; M, model group; SNP, sodium nitroprusside group. Scale bars: 50 μm. Original magnification ×200.

HE pathological results are shown in Figure 3. Obvious interstitial edema and enhanced infiltration of inflammatory cells were observed in both the embolic lung tissue and the non-embolic lung tissue.

### Expression of TLR4/NF-κB/HIF-1α/Gal-3 in embolism area

**FIGURE 4:**
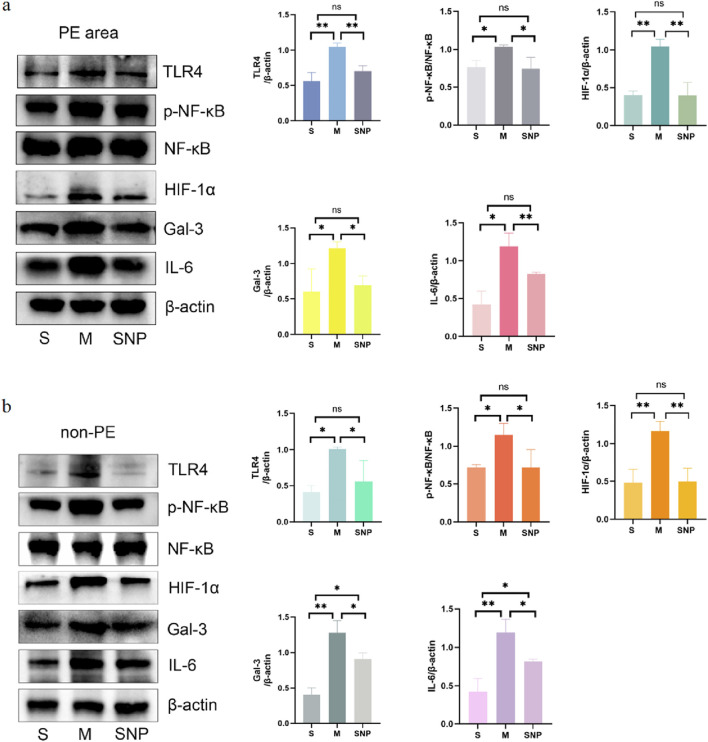
Protein expressions of HIF-1α, Gal-3, TLR4, p-NF-κB, NF-κB and IL-6 in pulmonary embolism **(a)** and non-pulmonary embolism **(b)** tissues detected by Western blot. Data are expressed as mean ± SD by bar graphs. S, sham operation group; M, model group; SNP, sodium nitroprusside group. PE, pulmonary embolism tissue; non-PE, non-pulmonary embolism tissue. ns *P >* 0.05, *P < 0.05, **P ≤ 0.01; ***P ≤ 0.001, ****P ≤ 0.0001.

**FIGURE 5:**
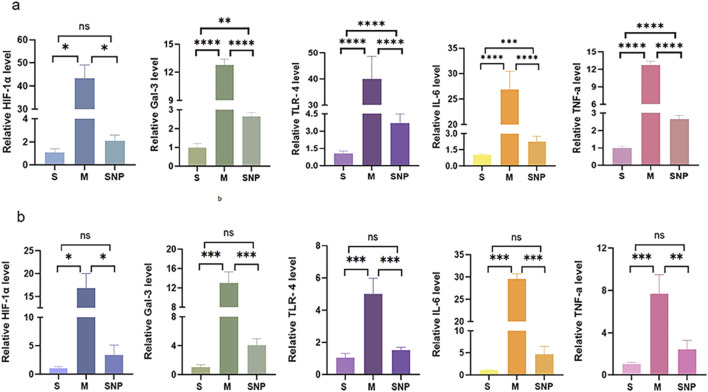
mRNA level of HIF-1α,Gal-3,TLR4,IL-6 and TNF-α in pulmonary embolism **(a)** and non-pulmonary embolism **(b)** tissues by RT-PCR. Bar graphs indicated that HIF-1α, Gal-3, TLR4, IL-6 and TNF-α, mRNA expressions of the M group in PE and non-PE tissues were significantly increased. HIF-1α, Gal-3, TLR4, IL-6 and TNF-α mRNA levels were significantly decreased than in the SNP group in PE and non-PE tissues. Data are expressed as mean ± SD by bar graphs. Ns *P >* 0.05, *P < 0.05, **P ≤ 0.01; ***P ≤ 0.001, ****P ≤ 0.0001. PE, pulmonary embolism tissue; non-PE, non-pulmonary embolism tissue. S, sham operation group; M, model group; group; SNP, sodium nitroprusside group.

**FIGURE 6:**
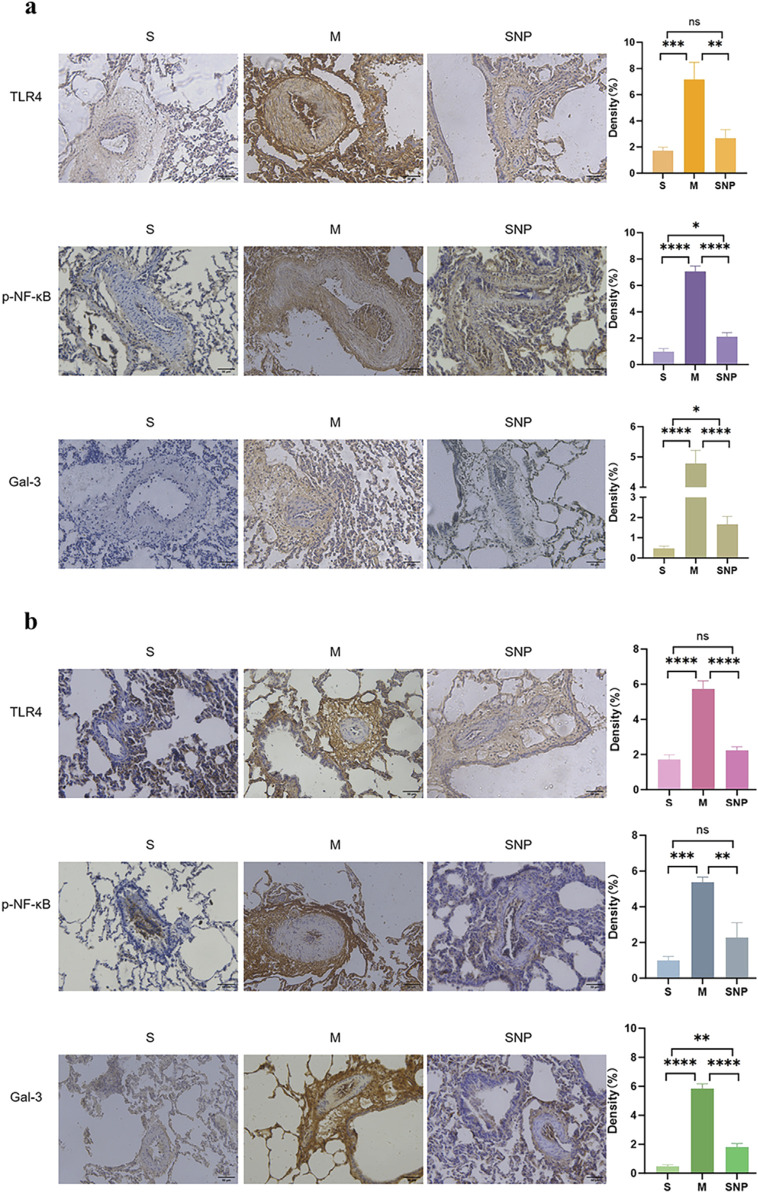
Gal-3, TLR4 and p-NF-κB features of pulmonary embolism **(a)** and non-pulmonary embolism **(b)** tissues. S, sham operation group; M, model group; SNP, sodium nitrprusside group, PE, pulmonary embolism tissue; non-PE, non-pulmonary embolism tissue. Gal-3, galectin-3; TLR4, toll-like receptor 4; p-NF-κB, phospho-nuclear factor κB. Scale bars = 200 µm. Original magnification×200. Ns *P >* 0.05, *P < 0.05, **P ≤ 0.01; ***P ≤ 0.001, ****P ≤ 0.0001, ****P ≤ 0.0001. Scale bars = 50 μm. Original magnification ×200.

In the acute pulmonary embolism combined with shock model, the TLR4/NF-κB/HIF-1α pathway in the embolism area might be activated, promoting the occurrence of inflammatory response, stimulating the production of downstream inflammatory factors such as IL-6 and TNF-α, and aggravating pulmonary circulatory failure. WB analysis and RT-qPCR showed that in the embolism area, the expression of TLR4, p-NF-κB, IL-6 and Gal-3 was significantly upregulated in the M group compared with the S group, which could be significantly downregulated by SNP intervention (Figures 4a, 5a). Immunohistochemical staining showed that compared with the S group, the expression of Gal-3 and TLR4 and the phosphorylation of NF-κB was increased in the M group. After treatment with SNP, the expression of Gal-3, TLR4 and p-NF-κB was reduced (Figure 6a).

### Expression of Gal-3/TLR4/NF-κB in non-embolized areas

Our experimental studies have shown that the activation of inflammatory mediators in the non-embolic area in the acute pulmonary embolism combined with shock model plays an important role in pulmonary blood flow failure. In the non-embolic area, compared with the S group, the protein and mRNA expression of HIF-1α, Gal-3, TLR4, p-NF-κB was significantly upregulated in the M group, which could be reversed by SNP (Figures 4b, 5b). Immunohistochemical staining showed similar results (Figure 6b).

### NO level in embolism and non-embolism tissue

**FIGURE 7:**
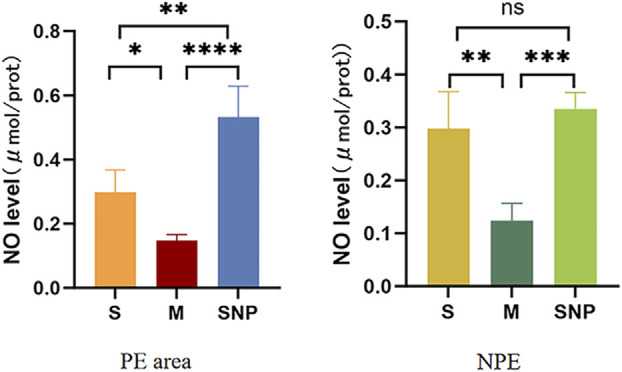
The expression level of nitric oxide in the pulmonary embolism and non-pulmonary embolism tissue. S, sham operation group; M, model group, SNP, sodium nitroprusside group. PE, pulmonary embolism tissue; NPE, non pulmonary embolism tissue. Ns *P >* 0.05, *P < 0.05, **P ≤ 0.01; ***P ≤ 0.001, ****P ≤ 0.0001, ****P ≤ 0.0001.

NO level was reduced in both embolism and non-embolism tissue in M group compared to S group, which could be reversed by SNP (Figure 7).

## Discussion

Our research results are as follows: 1) When acute pulmonary embolism combined with shock occurs, the lung tissue in the embolism area and non-embolism area is in a state of severe hypoxia, followed by the activation of the downstream TLR4/NF-κB/HIF-1α signaling pathway, HIF-1α further activates Gal-3 expression. When acute pulmonary embolism occurs, the pulmonary vessels in the embolized and non-embolized areas undergo excessive spasm, damaging the vascular endothelium and severely reducing the expression of NO. The inflammatory response is overly activated, which further aggravates the damage to the vascular endothelium, leading to a sustained decrease in NO release and ultimately causing circulatory failure. 2) In this acute pulmonary embolism combined with shock model, SNP (0.35 mg/kg) treatment enhances NO release, and therefore reduces pulmonary vasospasm in the embolism area and non-embolism area, possibly through inhibiting the expression of TLR4/NF-κB/HIF-1α signaling pathway, thus breaking the vicious cycle of circulatory failure, relieving shock.

### Changes of TLR4/NF-κB/HIF-1α signaling pathway and Gal-3 in rabbit model of APE combined with shock

It is well known that high-risk acute pulmonary embolism is often featured with high mortality due to shock, hypoxemia, and right heart failure. Hypoxia can trigger acute and chronic inflammation responses, vascular endothelial injury, metastasis, and apoptosis, forming a vicious cycle of circulatory failure, jointly responsible for the poor prognosis of the disease. Our experiments indicate that in the acute pulmonary embolism combined with shock model, the TLR4/NF-κB/HIF-1α signaling pathway is activated, which might stimulate the upregulation on the expression of downstream Gal-3, and the release of inflammatory factors such as IL-6 and TNF-α. Enhanced inflammatory responses is a known pathogenic factor of vascular endothelial injury, NO release reduction, and pulmonary vasospasm aggravation in both the embolic and non-embolic areas, forming a vicious cycle of circulatory failure. Our results are in line with a previous report confirming that hypoxic pulmonary hypertension injury could be aggravated by activating the TRAF6/NF-κB/HIF-1α pathway (Wang et al., 2023). HIF-1α is one of the most important factors in the pathogenesis of acute lung injury. It participates in the occurrence of multiple reactions in acute lung injury, targets and binds to the core DNA sequence in the promoter HRE, and participates in the activation of genes of multiple pathogenesis such as vascular endothelial injury and inflammation (Yeh et al., 2011; Semenza, 2014; Suresh et al., 2023). Accumulating evidenes sugget that HIF-1α is not only a factor that causes hypoxia adaptation, but also involved in the occurrence of inflammation and oxidative stress responses under severe hypoxia (Liu et al., 2020). Overexpression of HIF-1α can induce increased expression of Gal-3, and Gal-3 expression depends on the production of HIF-1α (Greijer et al., 2005). Gal-3 is a member of the galactoside-binding lectin superfamily and is involved in the development of many diseases such as inflammation (Bouffette et al., 2023), vascular endothelial injury (Pang et al., 2023), and atherosclerosis (Gao et al., 2020). Previous studies have shown that Gal-3 is involved in regulating the production of proinflammatory cytokines, and inhibition of Gal-3 leads to reduced expression of proinflammatory cytokines such as IL-6 and Interleukin-1β (IL-1β) (Chen et al., 2015). Gal-3 is also closely related to oxidative stress response by inducing oxidative stress in neutrophils, monocytes, mast cells and other cells by producing superoxide anion (Seropian et al., 2023). In pulmonary hypertension, Gal-3 activates the AKT signaling pathway to overactivate pulmonary vascular adventitial fibroblasts, promoting the occurrence and development of PAH (pulmonary arterial hypertension) (Barman et al., 2019). Similarly, our experiment verifies that severe spasm of the pulmonary artery might damage the vascular endothelial cells, reduce the release of NO, activate the expression of TLR4/NF-κB pathway as well as Gal-3 in the acute pulmonary embolism combined with shock model. Numerous literature reports that elevated levels of Gal-3 are associated with poor prognosis and mortality of cardiovascular and cerebrovascular diseases such as pulmonary arterial hypertension, ARDS, acute myocardial infarction, and stroke (Tsai et al., 2012; Calvier et al., 2016; Hara et al., 2020; Sayed et al., 2022), and hemodynamic instability in the acute phase of acute pulmonary embolism is one of the main causes of short-term death, and irreversible shock leads to death in high-risk patients with pulmonary embolism (Quezada et al., 2019). Our experiments showed that when acute pulmonary embolism occurs in shock, Gal-3 is significantly upregulated, so we propose that Gal-3 may serve as a biomarker indicating the high mortality risk in the setting of acute pulmonary embolism with shock.

### Effect and mechanism of SNP on rabbit model of APE combined with shock

**FIGURE 8:**
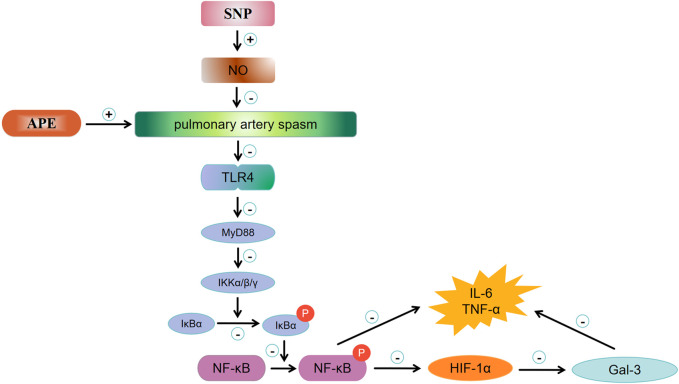
Mechanism of MPE and potential working effects of SNP. APE, acute pulmonary enbolism; SNP, sodium nitroprusside group; NO, nitric oxide; TLR4, toll-like receptor 4; MyD88, myeloid differential protein-88; IKK, inhibitor of kappa B kinase; IKB,I kappa B kinase; p-NF-κB, phosphor-nuclear factor κB; HIF-1α, hypoxia inducible factor 1 alpha; Gal-3, galectin-3.

SNP is a fast-acting vasodilator. Lim et al. previously demonstrated that SNP can reduce pulmonary circulation resistance, systemic vascular resistance and cardiac filling pressure, improve exercise capacity and prognosis in patients with mixed pulmonary hypertension and left heart disease (Verbrugge et al., 2015; Lim and Zaphiriou, 2016). Our previous studies showed that SNP treatment could effectively improve pulmonary blood flow, reduce pulmonary artery pressure, and reverse circulatory failure, which indicates that the dilation effect of SNP on pulmonary vasculature is far greater than its hypotensive effect on peripheral circulation in the rabbit model of massive pulmonary embolism (MPE) (Yu et al., 2018). In addition, SNP treatment reduced the expression of sympathetic mediators TH (Tyrosine hydroxylase) and NPY (neuropeptide Y) in lung tissues in both embolic and non-embolic areas, which helps to alleviate the vicious cycle of hypoxemia, pulmonary vasospasm and sympathetic nerve activation. In addition to the direct effect of sympathetic nerve activity, NO released by the pulmonary vascular endothelium might also play an important role in SNP’s reversal of acute pulmonary embolism combined with shock (Wang et al., 2019). NO is a relaxing factor of endothelial cells. Increased NO synthesis allows blood vessels to maintain a vasodilation state. Related literature indicates that inhaled NO can rapidly dilate the pulmonary artery and has good application prospects in the treatment of pulmonary hypertension (Hayward et al., 2001). Our study showed that TLR4 expresion was reduced post SNP in the lung tissues of this model. Since TLR4 is not a direct receptor of SNP, the effects of SNP on TLR4 expression might be indirect. Accordingly, a previous study on sickle cell anemia demonstrated that the use of sodium nitroprusside could indirectly inhibit the TLR4 activation and reduce inflammatory responses and ROS (reacive oxygen species) levels (Nader et al., 2020). Our previous studies have confirmed that SNP treatment can rapidly increase the expression of NO in blood vessels, significantly dilate blood vessels, improve tissue perfusion, improve the body’s hypoxia state, reduce oxidative stress and inflammatory damage in the body, and partially reverse acute circulatory failure (Wang et al., 2019). Other studies also are in line with previous findings showing that sodium nitroprusside could inhibit the activation of multiple inflammatory signaling pathways and reduce the release of inflammatory cytokines (Chen et al., 2023; Seoane et al., 2024). Our study further demonstrates that after SNP treatment, the expressions of the TLR4/NF-κB/HIF-1α signaling pathway and Gal-3 in the embolized area and the non-embolized area were downregulated, inflammation damage was alleviated, and ultimately, resulting in reversed circulatory failure. This may be due to the fact that SNP promotes the release of NO by vascular endothelium, alleviates vascular spasm throughout the lungs, and reduces the release of downstream inflammatory factors. In summary, Present study results expanded the working mechanism of SNP in the rabbit acute pulmonary embolism combined with shock model, showing that the activation of the TLR4/NF-κB/HIF-1α signaling pathway in the embolic and non-embolic areas of lung tissue might lead to enhanced release of downstream inflammatory factors and upregulated expression of Gal-3 (Figure 8). Excessive inflammatory response might further stimulate the pulmonary vascular endothelium dysfunction, causing a decrease in endogenous NO release, leading to aggravated pulmonary vasospasm in the embolic and non-embolic areas, and ultimately resulting in the circulatory failure. SNP treatment can release the vasodilator factor NO in the embolic and non-embolic areas, and reduce pulmonary vasospasm, which can inhibit the expression of the TLR4/NF-κB/HIF-1α signaling pathway in the embolic and non-embolic areas, downregulate Gal-3 and reduce the damage of inflammation. All mentioned above finally restore blood flow in lung tissue, and reverse the shock state. In addition, we propose that Gal-3 may be a useful biomarker for risk stratification in patients with acute pulmonary embolism combined with shock.

## Data Availability

The original contributions presented in the study are included in the article/Supplementary Material, further inquiries can be directed to the corresponding authors.
