# Supplementary material for: Sodium nitroprusside improves circulatory failure in rabbit acute pulmonary embolism combined with shock model possibly by enhancing NO release and inhibiting TLR4/NF-кB/HIF-1α signaling pathway
Source: Front Physiol. 2025 Jul 1;16:1573405. doi: 10.3389/fphys.2025.1573405 (PMC12259664; doi:10.3389/fphys.2025.1573405)

PE area

TLR4

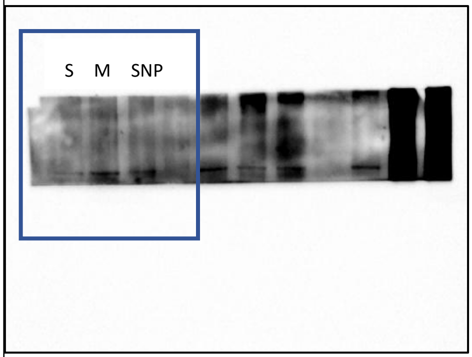

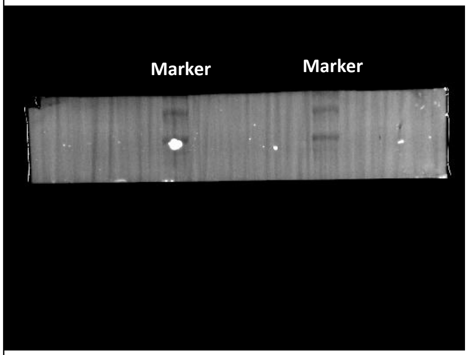

200kDa
140kDa
110kDa
90kDa

Merge

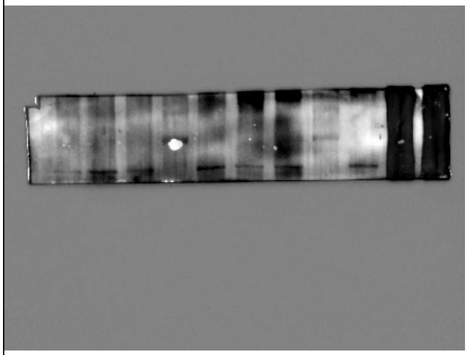

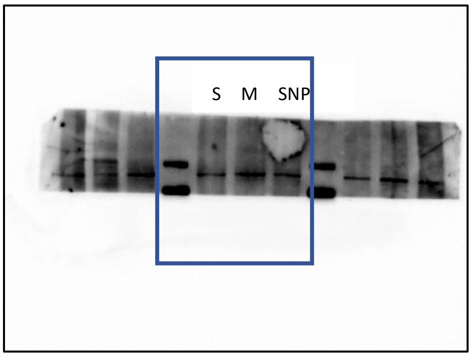

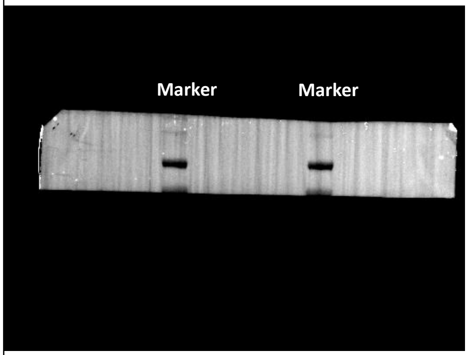

140kDa
110kDa
90kDa

Merge

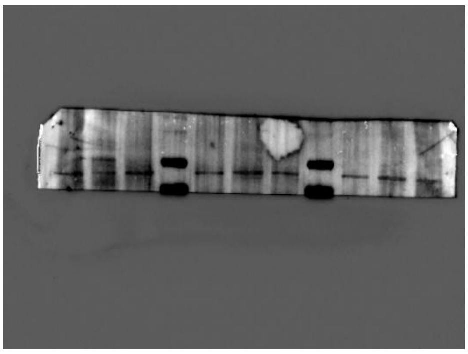

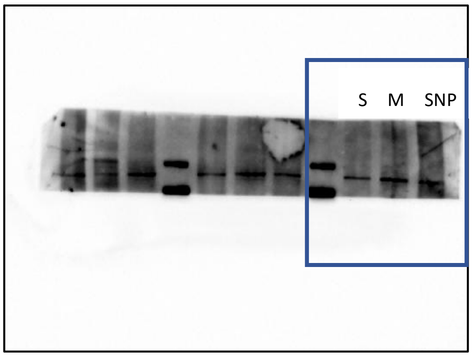

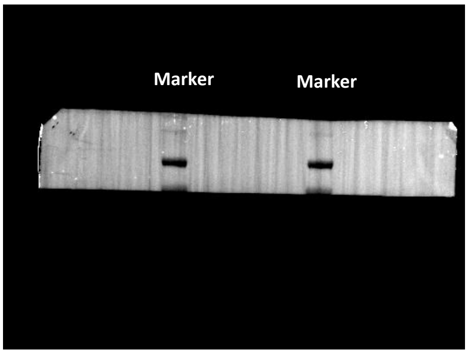

140kDa
110kDa
90kDa

Merge

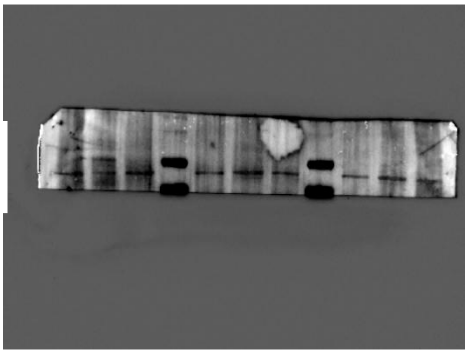

PE area

p-NF- κ B

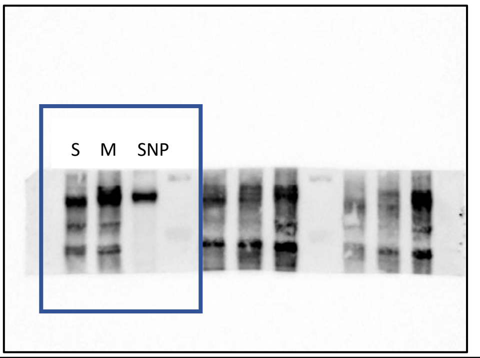

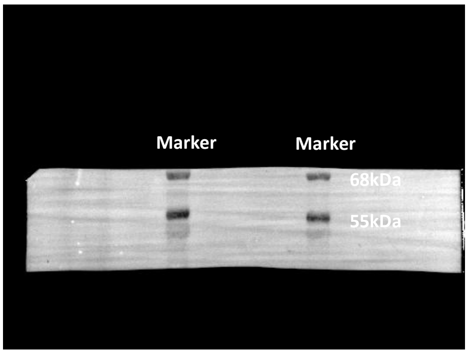

Merge

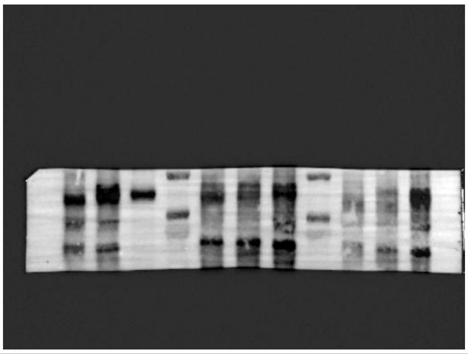

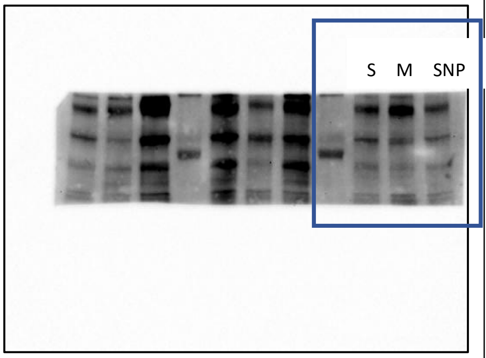

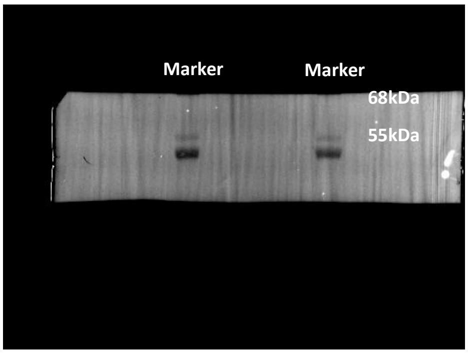

Merge

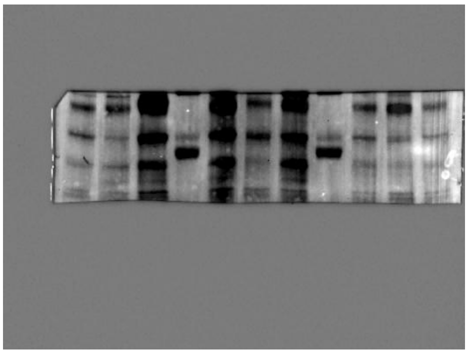

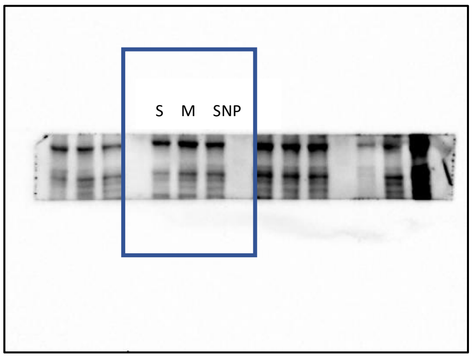

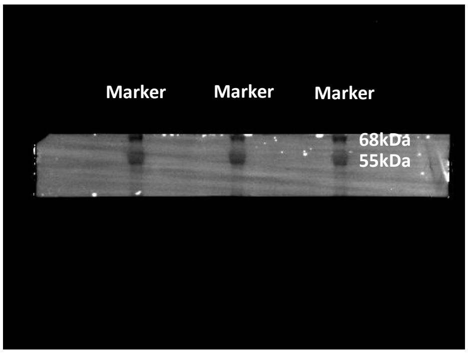

Merge

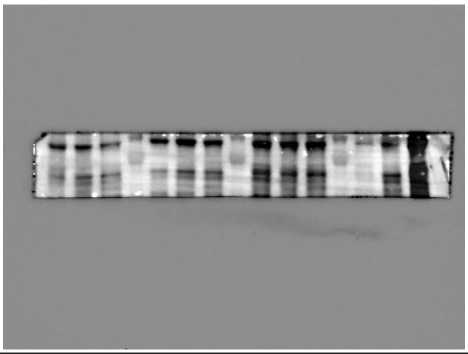

PE area

NF-κB

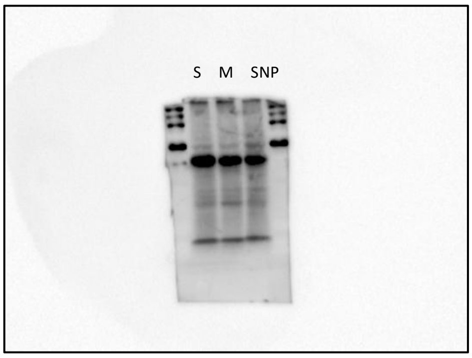

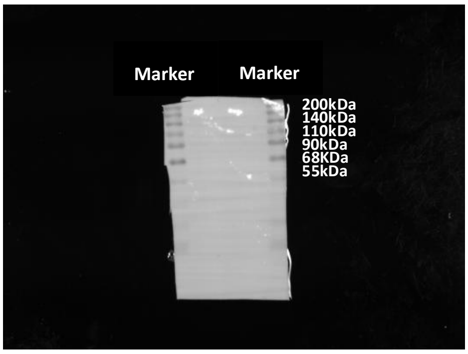

Merge

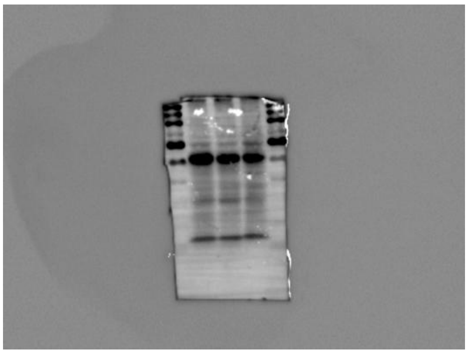

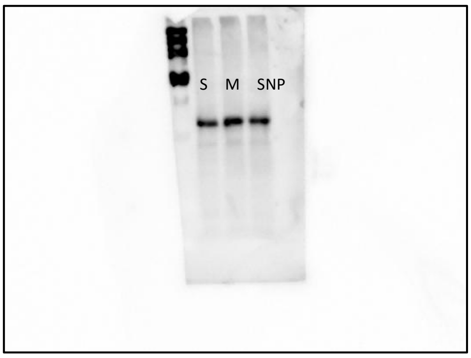

Marker

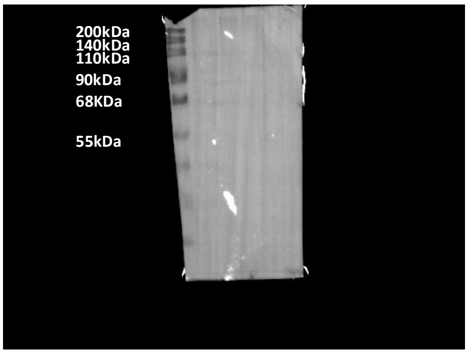

Merge

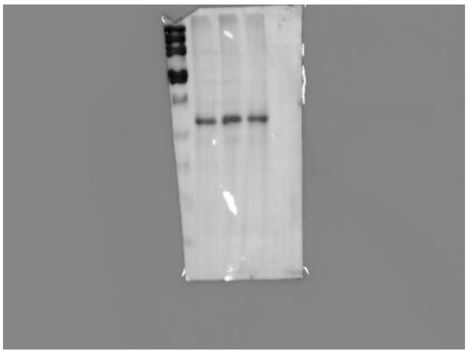

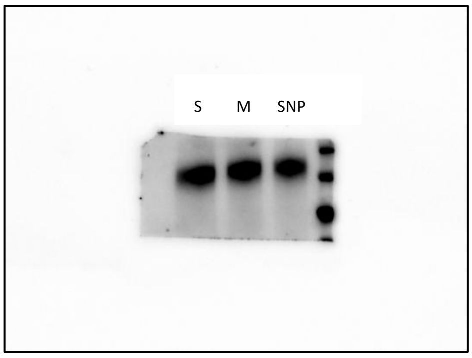

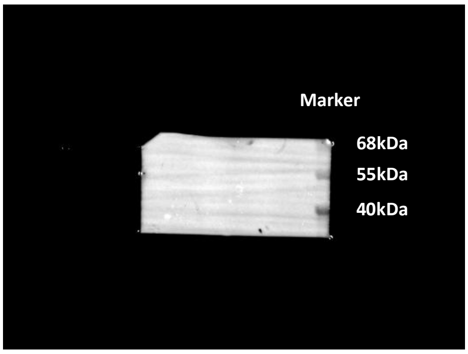

Merge

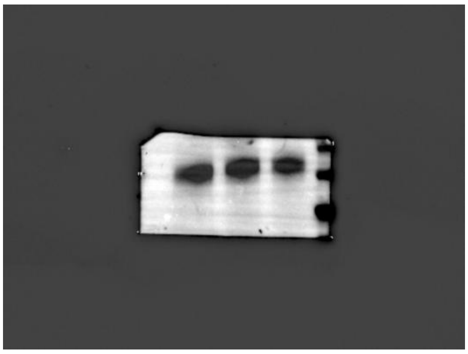

PE area

HIF-1α

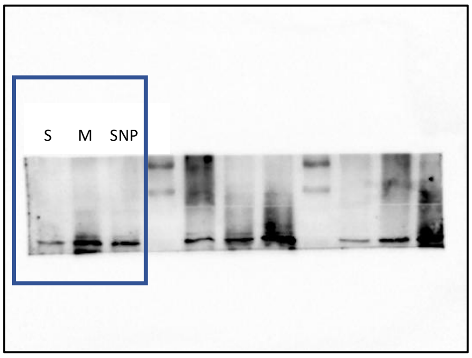

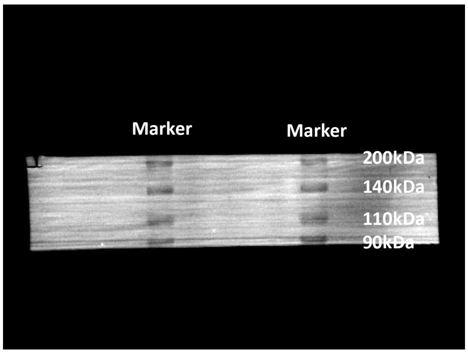

Merge

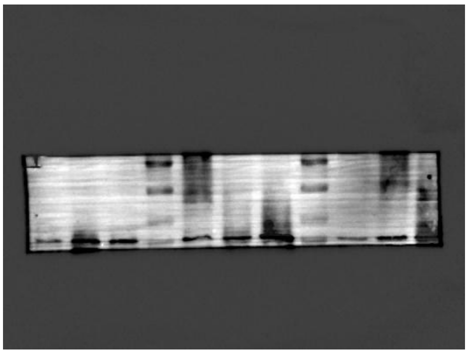

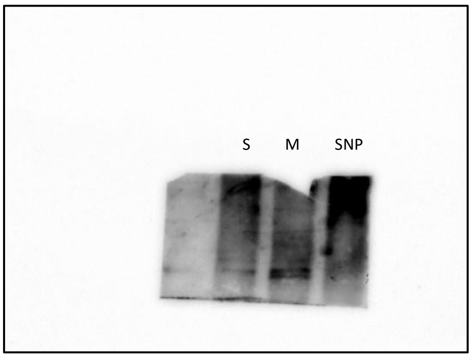

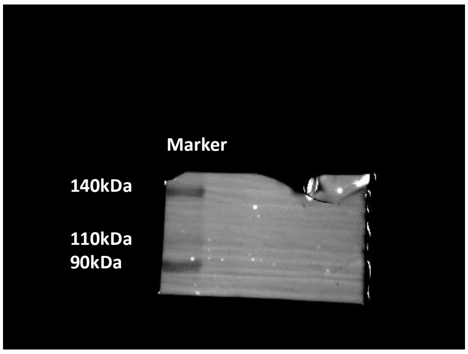

Merge

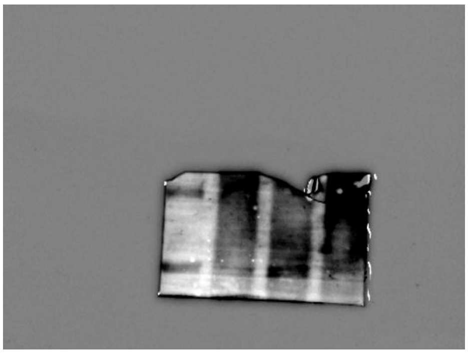

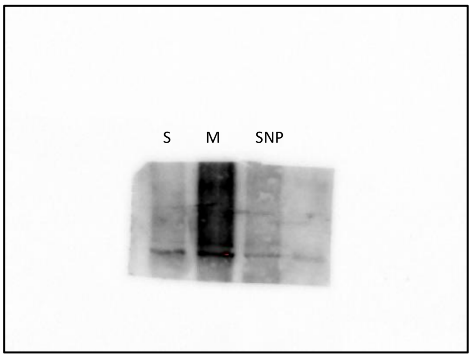

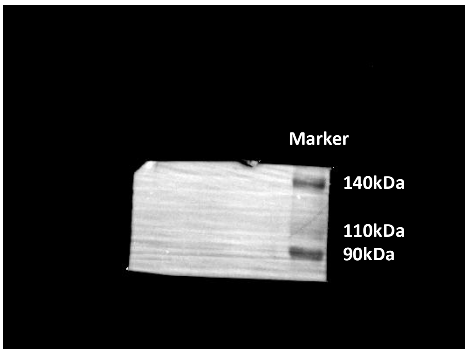

Merge

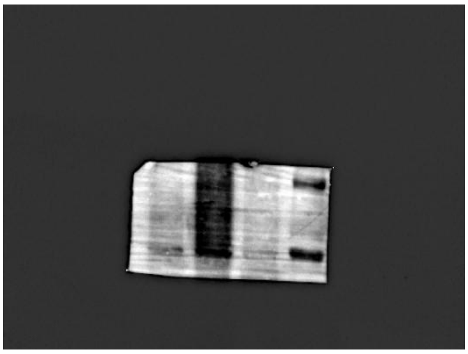

PE area

Gal-3

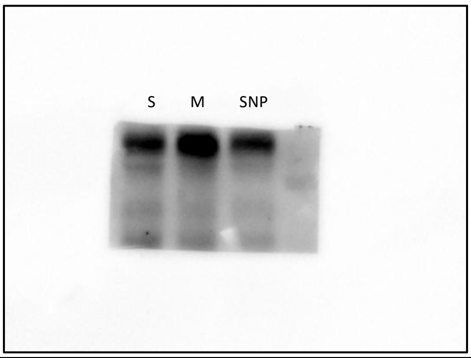	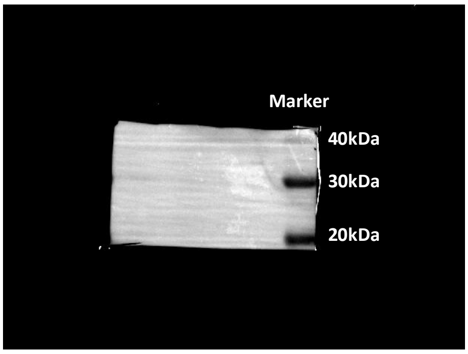	Merge 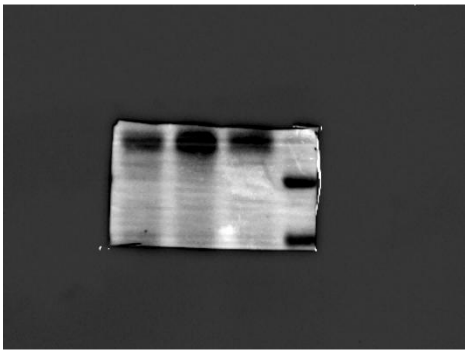
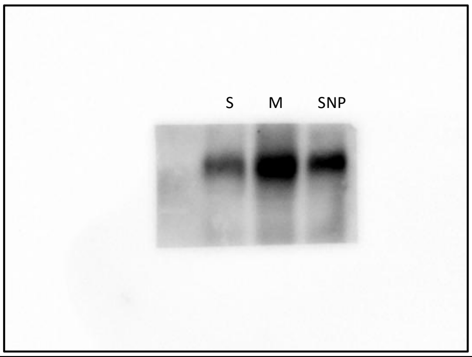	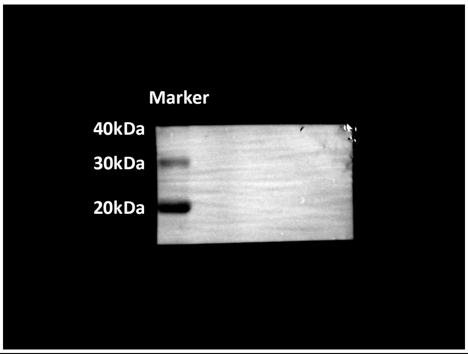	Merge 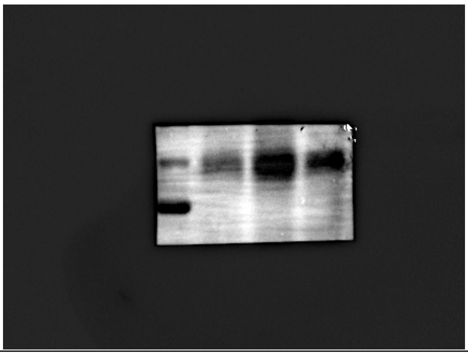
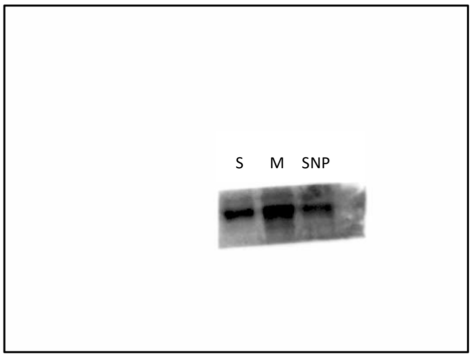	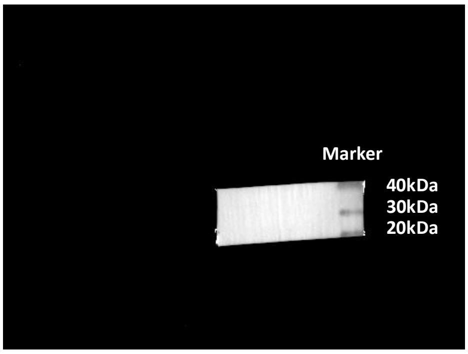	Merge 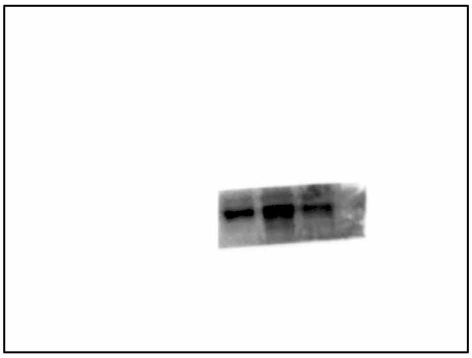

PE area

IL-6

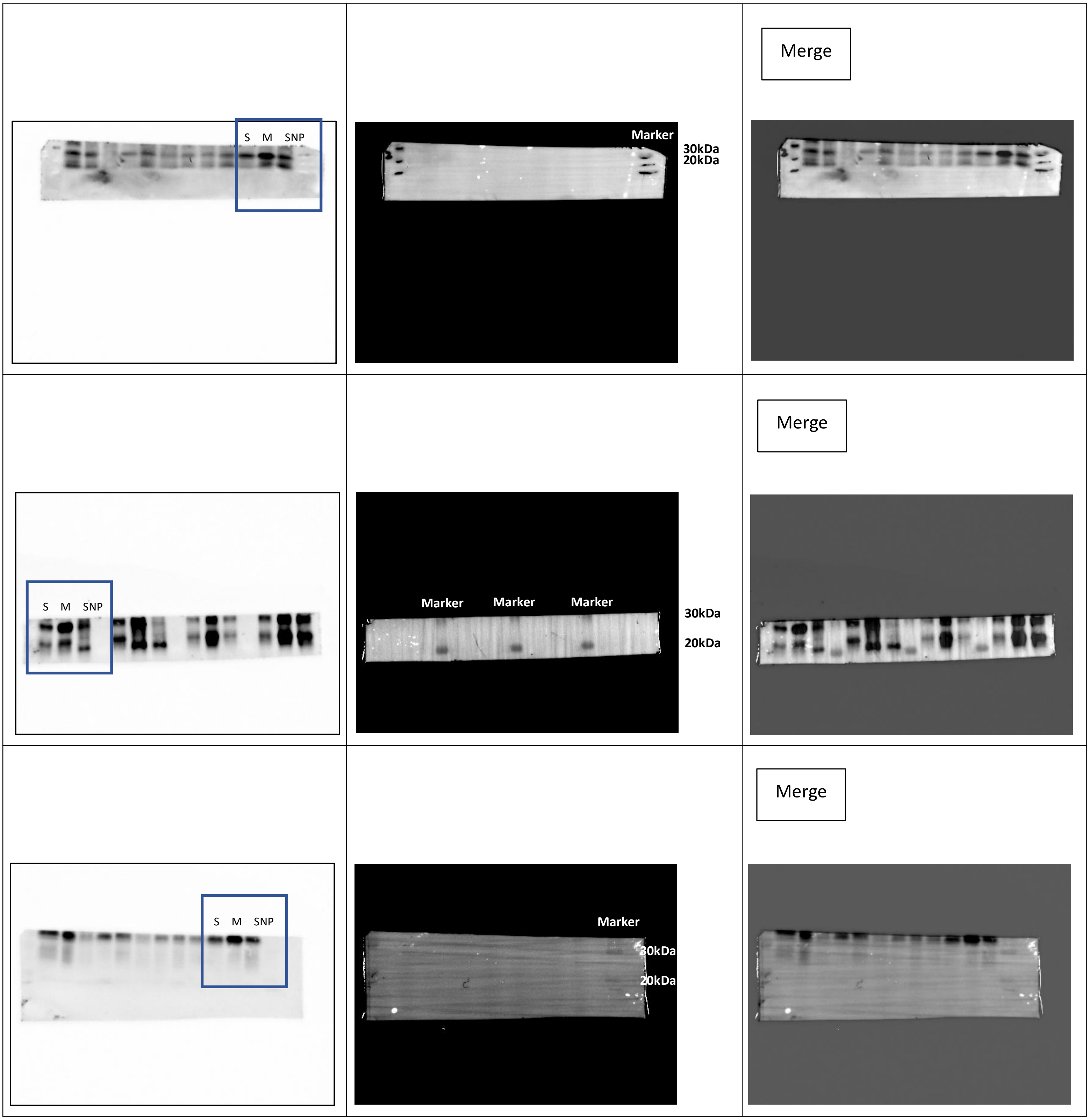
S M SNP
Marker
30kDa
20kDa
Merge
S M SNP
Marker Marker Marker
30kDa
20kDa
Merge
S M SNP
Marker
30kDa
20kDa
Merge

PE area

β-actin

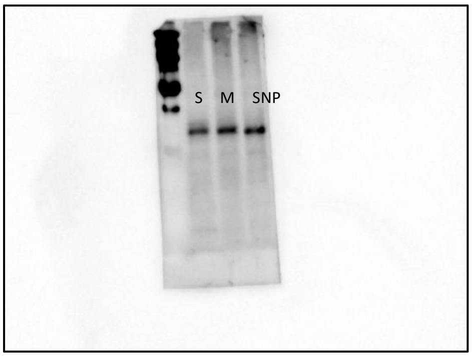

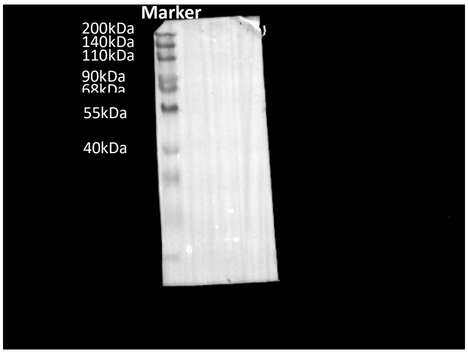

Merge

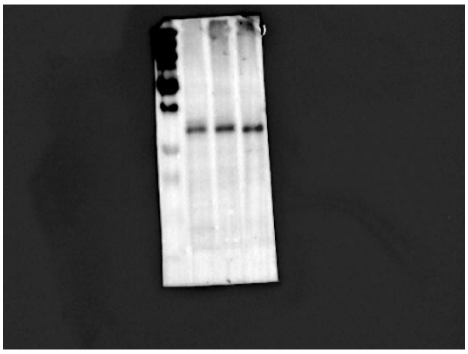

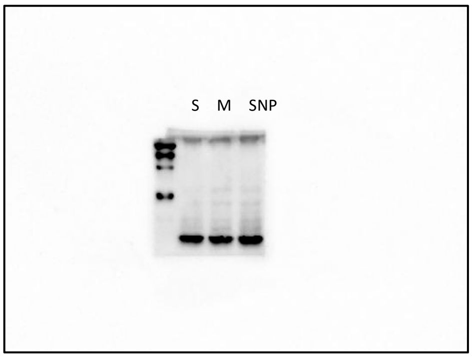

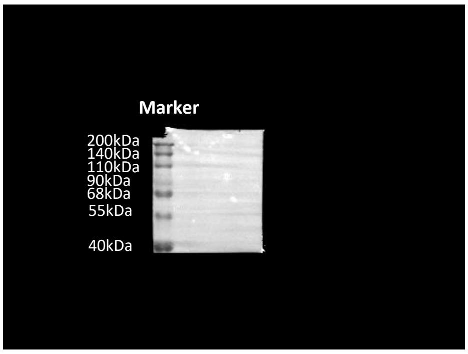

Merge

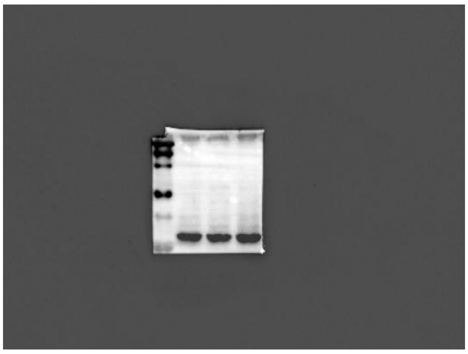

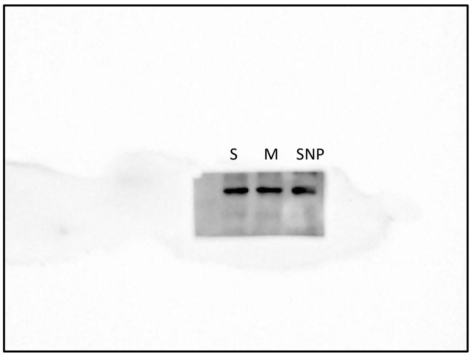

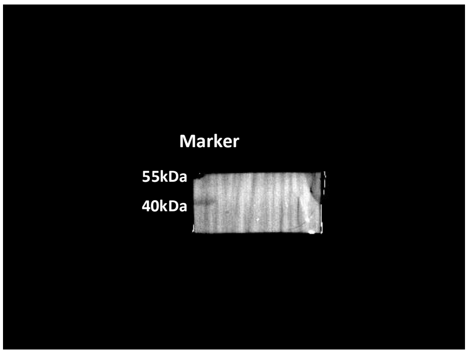

Merge

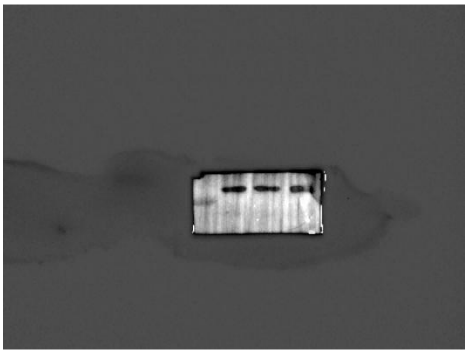

NPE area

TLR4

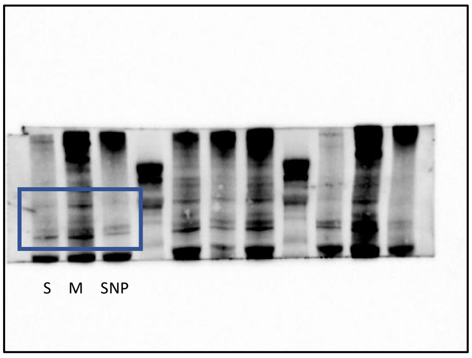

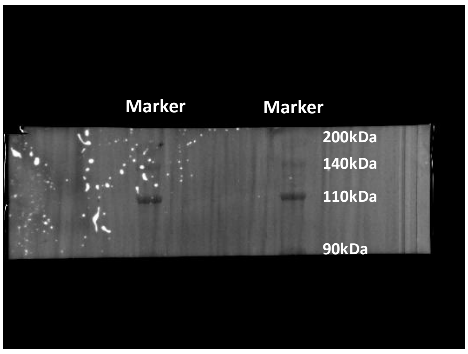

Merge

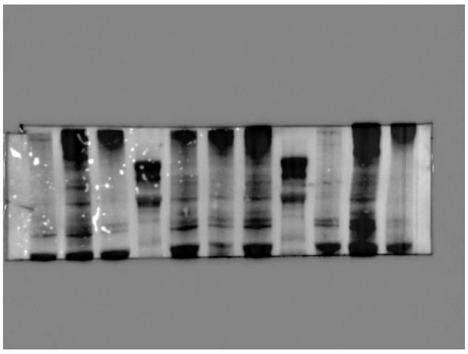

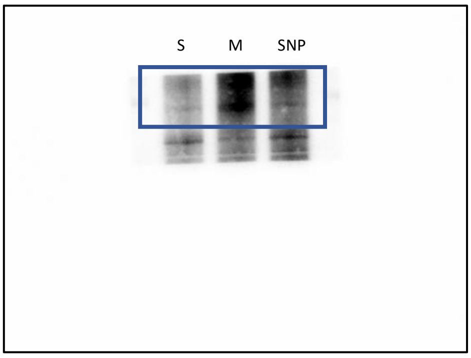

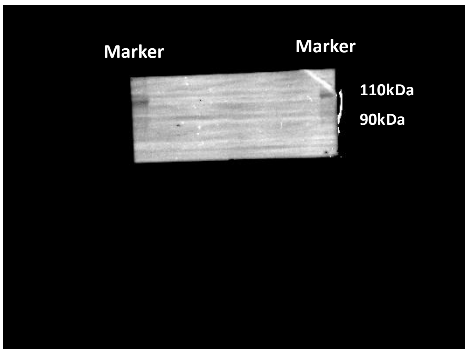

Merge

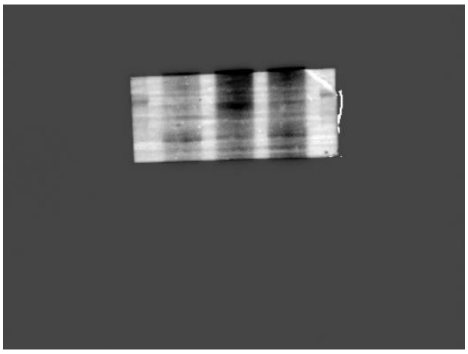

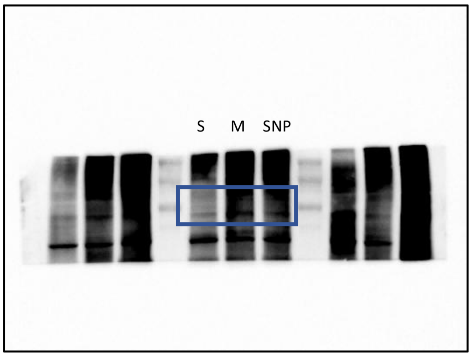

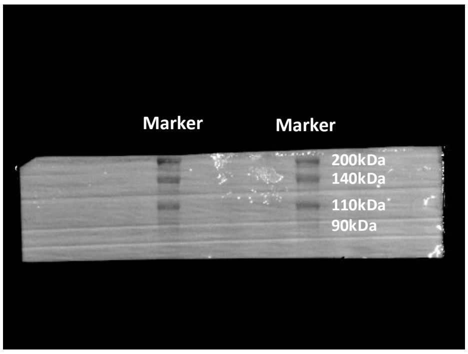

Merge

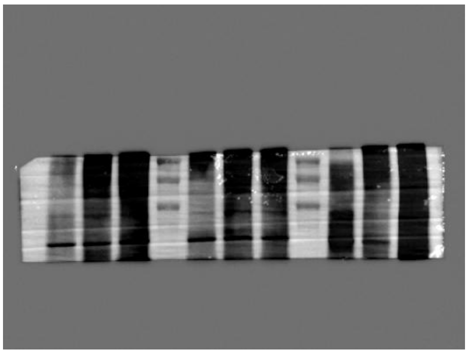

NPE area

p-NF- κ B

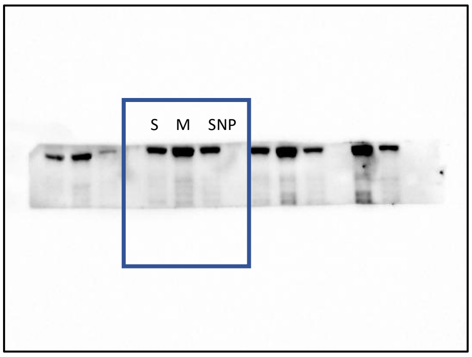

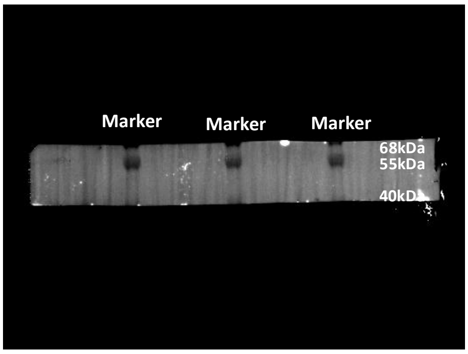

Merge

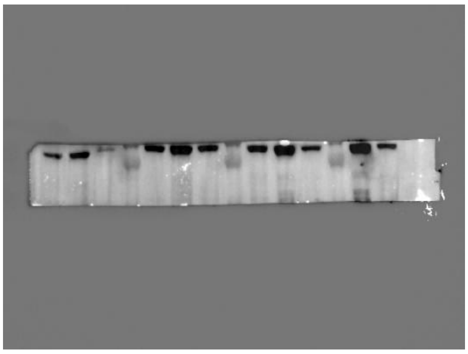

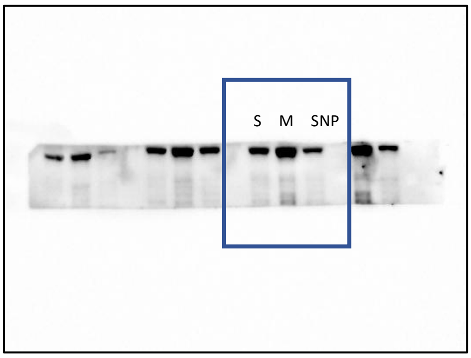

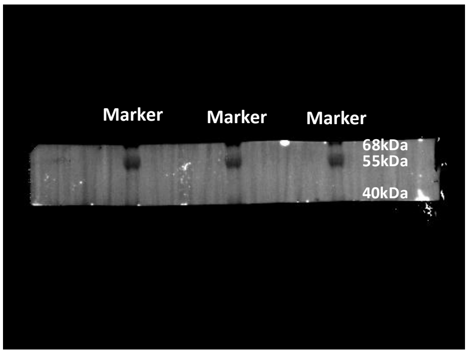

Merge

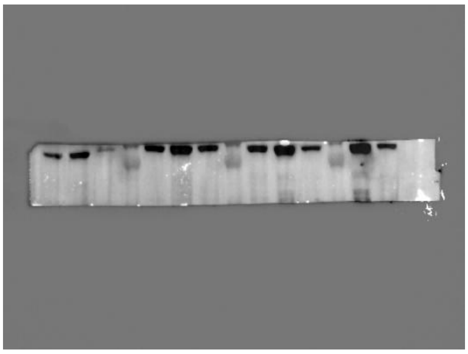

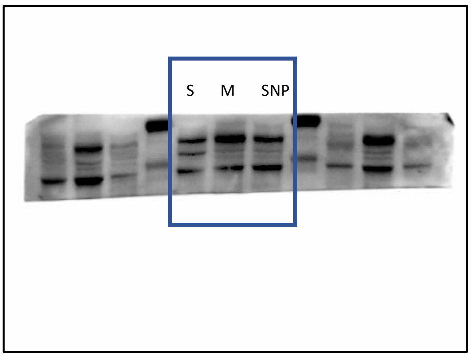

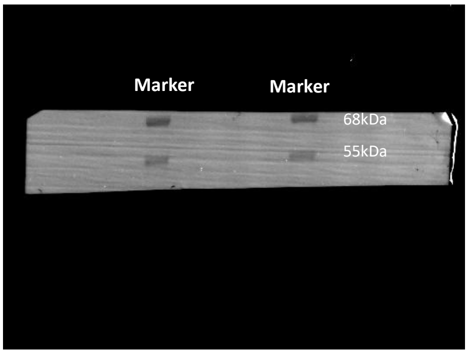

Merge

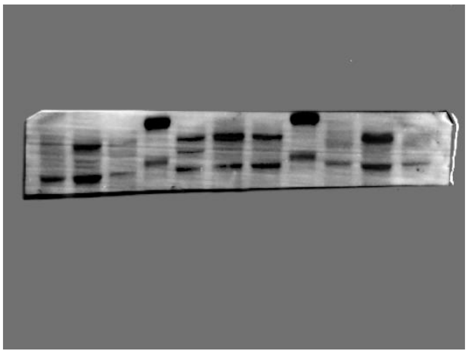

NPE area

NF- κ B

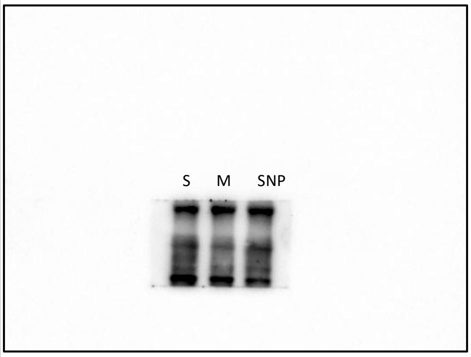

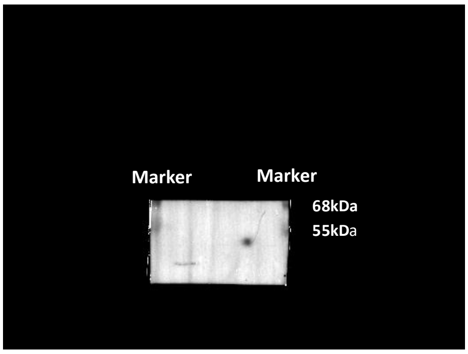

Merge

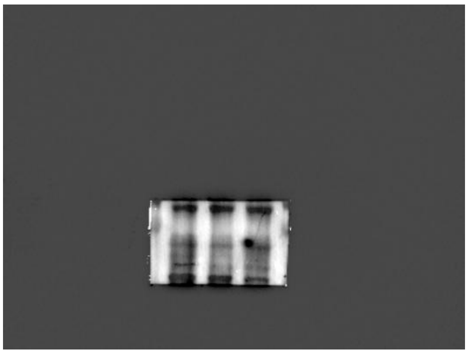

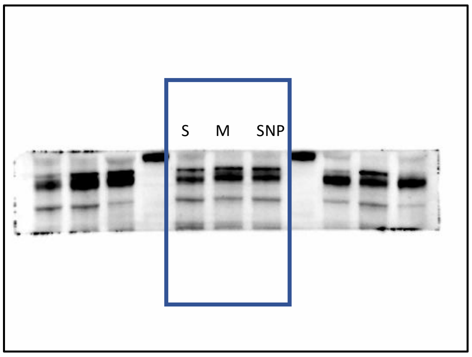

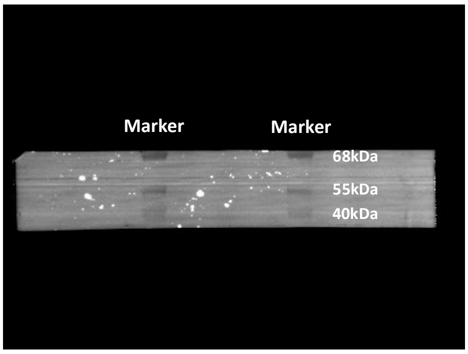

Merge

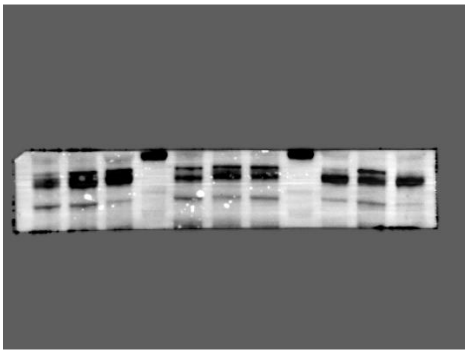

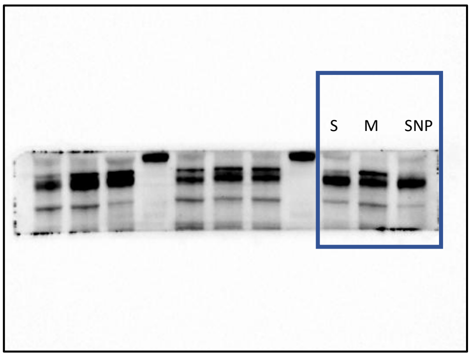

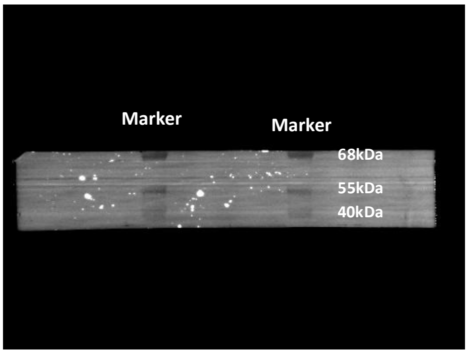

Merge

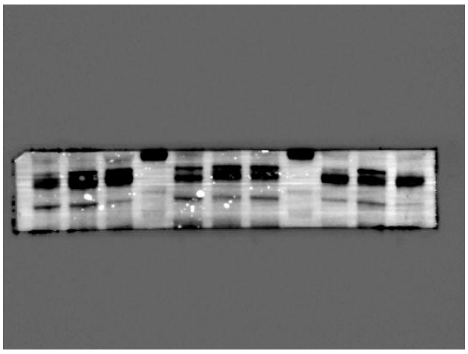

NPE area

HIF-1 α

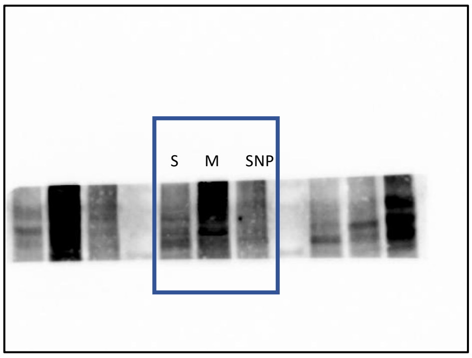

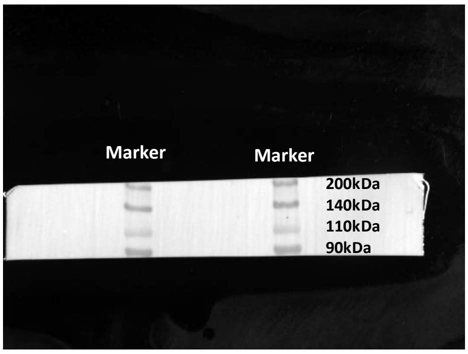

Merge

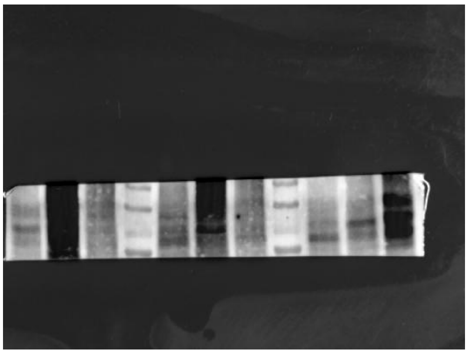

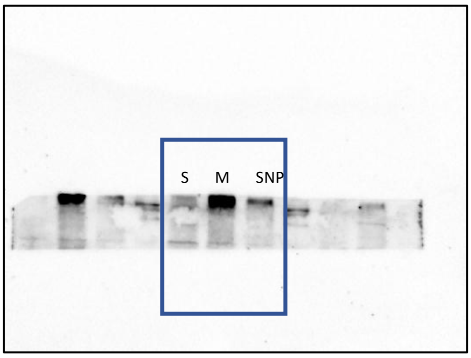

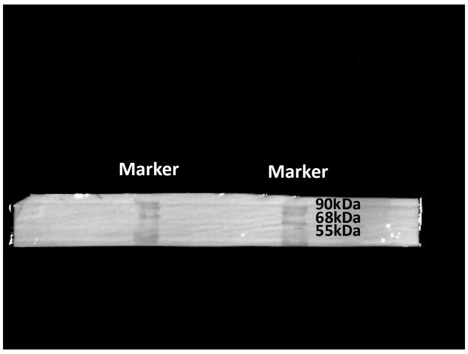

Merge

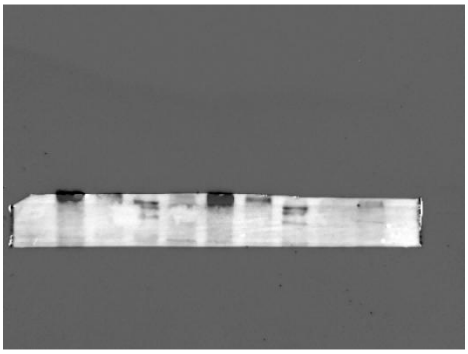

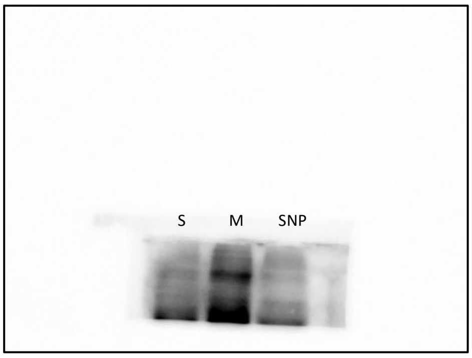

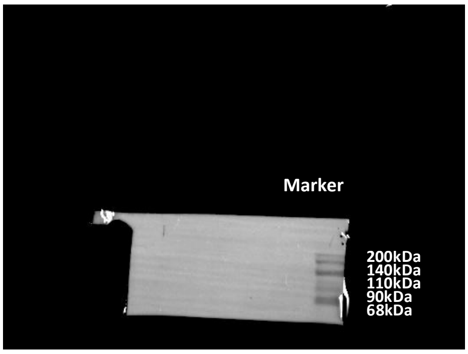

Merge

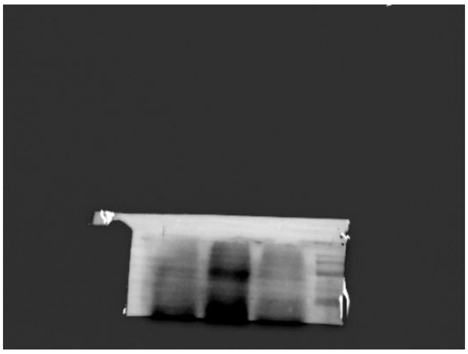

NPE area

Gal-3

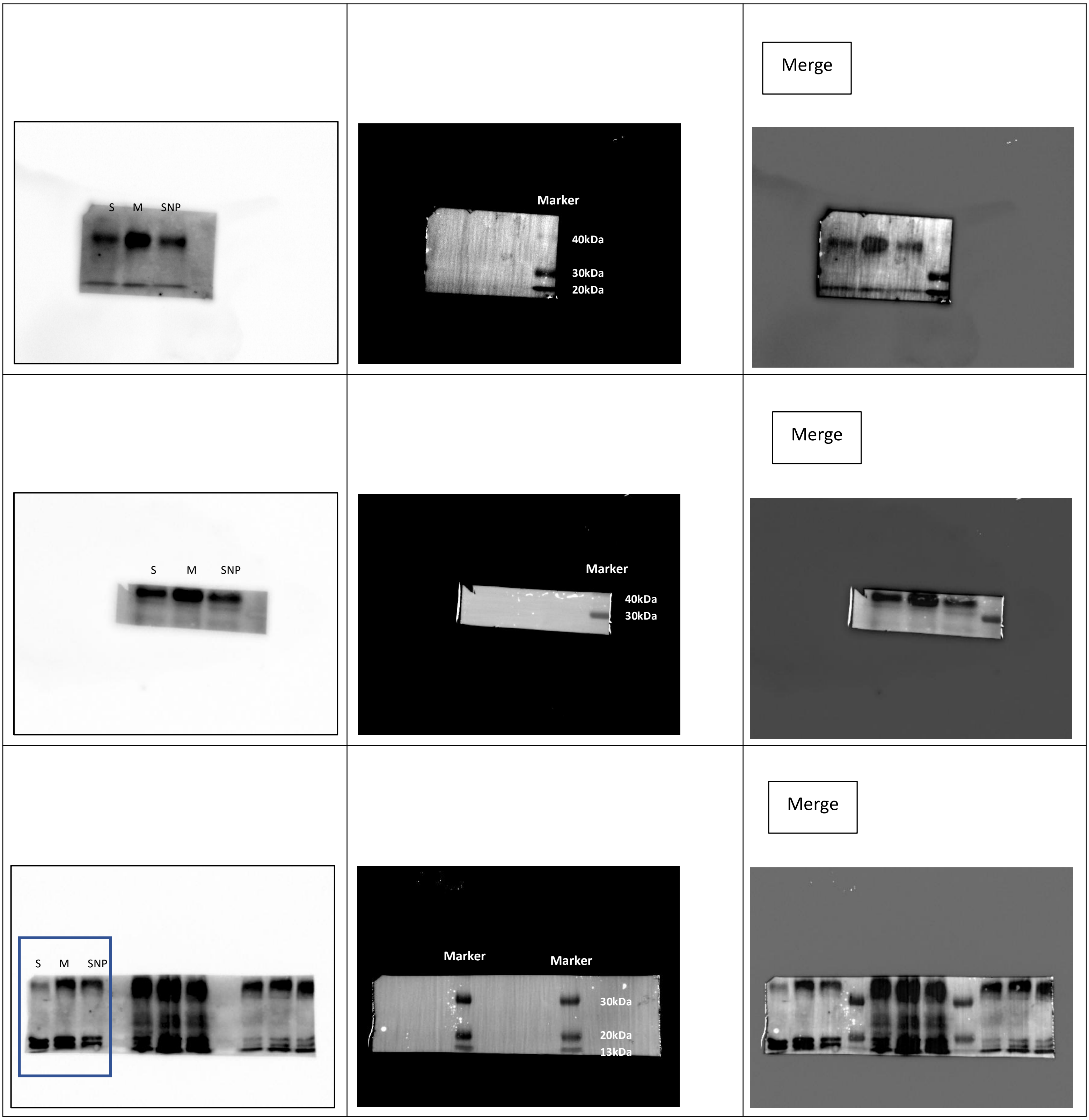

NPE area

IL-6

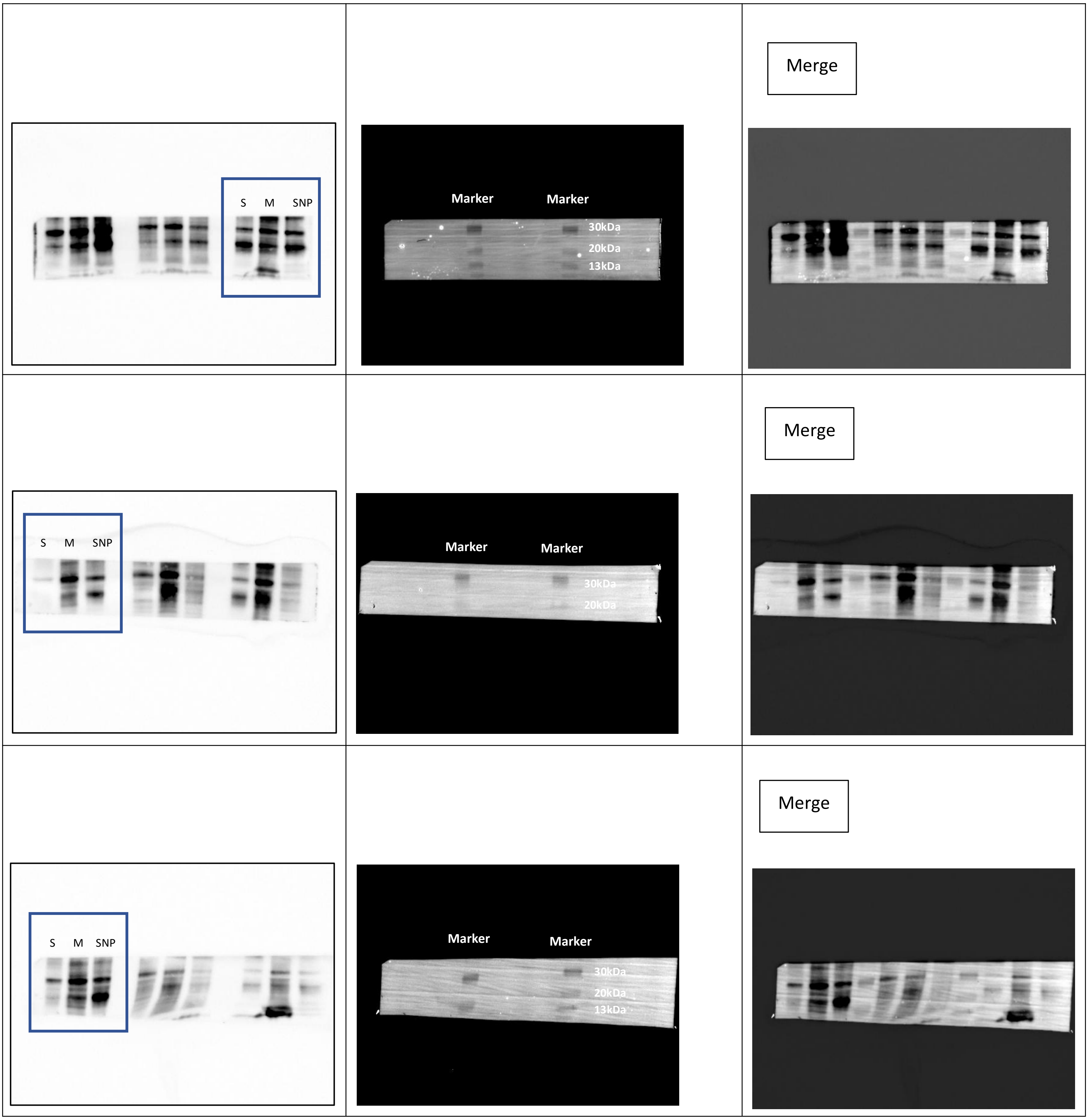

Merge
S
M
SNP
Marker
Marker
30kDa
20kDa
13kDa
Merge
S
M
SNP
Marker
Marker
30kDa
20kDa
Merge
S
M
SNP
Marker
Marker
30kDa
20kDa
13kDa

NPE area

β-actin

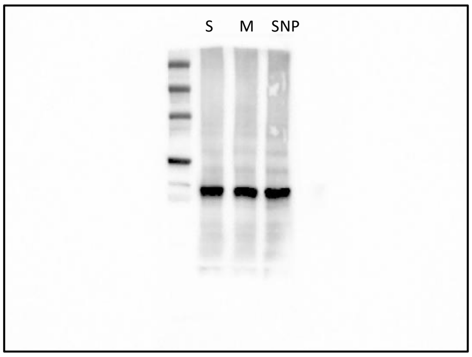

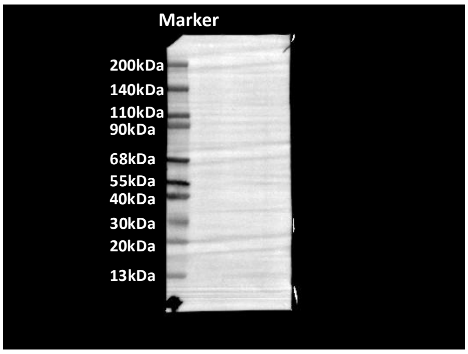

Merge

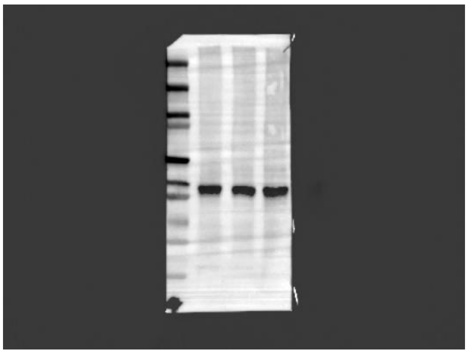

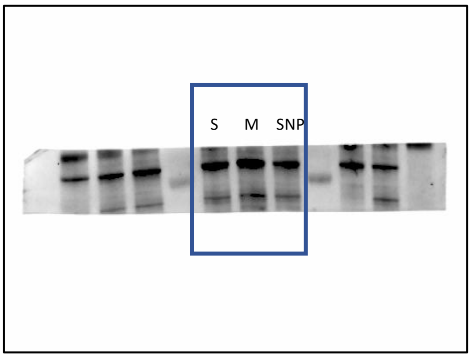

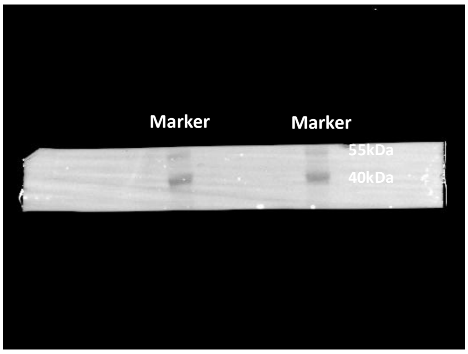

Merge

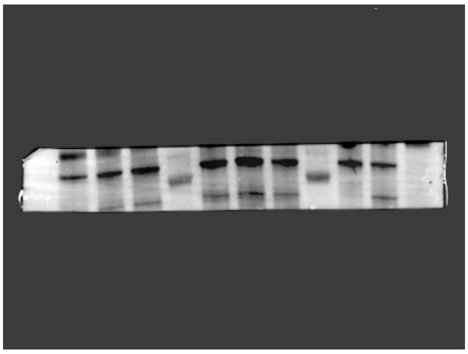

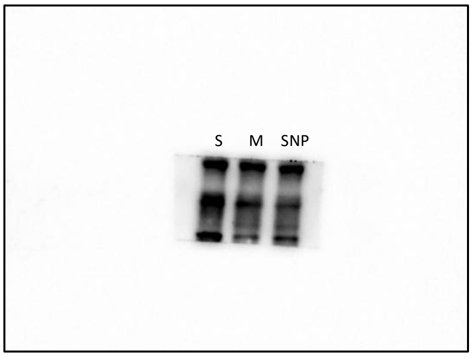

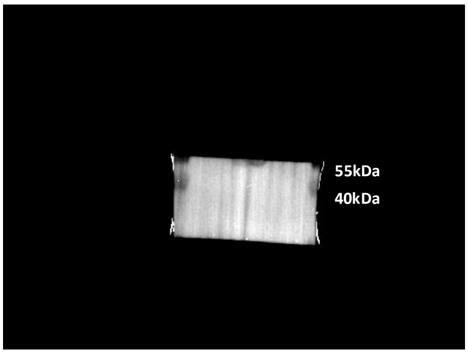

Merge

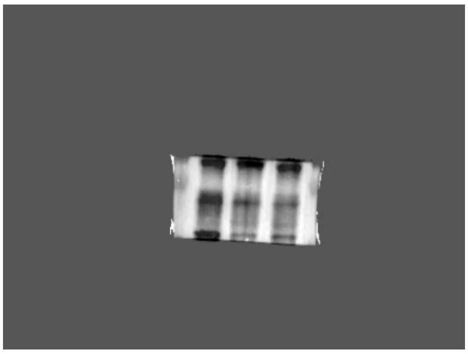

Supplement: Supplementary file 1 [file DataSheet2.pdf]
