# Supplementary material for: Sodium nitroprusside improves circulatory failure in rabbit acute pulmonary embolism combined with shock model possibly by enhancing NO release and inhibiting TLR4/NF-кB/HIF-1α signaling pathway
Source: Front Physiol. 2025 Jul 1;16:1573405. doi: 10.3389/fphys.2025.1573405 (PMC12259664; doi:10.3389/fphys.2025.1573405)
Supplement: Supplementary file 2 [file Table1.docx]

Supplementary Tables S1

Antibody information1

| Primary antibody | Brand | catalog numbers | lot numbers |
| --- | --- | --- | --- |
| HIF-1α | Wanleibio | WL01607 | T08231607 |
| TLR4 | Santa | SC-293072 | L0623 |
| p-NF-κB | Abmart | TP56372F | 10193199 |
| NF-κB | Bioss | Bsm-33117m | BD01102585 |
| Galectin-3 | Abcam | Ab2785 | GR294612-2 |
| IL-6 | Abcam | ab6672 | GR106736 |
| β-actin | Jackon | ANT321s | 30280307 |
