# Supplementary material for: Sodium nitroprusside improves circulatory failure in rabbit acute pulmonary embolism combined with shock model possibly by enhancing NO release and inhibiting TLR4/NF-кB/HIF-1α signaling pathway
Source: Front Physiol. 2025 Jul 1;16:1573405. doi: 10.3389/fphys.2025.1573405 (PMC12259664; doi:10.3389/fphys.2025.1573405)
Supplement: Supplementary file 4 [file Table2.docx]

Supplementary Tables S2

Antibody information2

| Primary antibody | Brand | catalog numbers | lot numbers |
| --- | --- | --- | --- |
| β-actin | Jackon | ANT321s | 30280307 |

| Secondary antibody | Brand | Catalog numbers | lot numbers |
| --- | --- | --- | --- |
| HRP Goat anti Rabbit lgG(HL) | Jackon | ANT020 | 1677860415 |
| HRP Goat anti mouse lgG(HL) | Jackon | ANT019 | 1679760320 |
