# Supplementary material for: Sodium nitroprusside improves circulatory failure in rabbit acute pulmonary embolism combined with shock model possibly by enhancing NO release and inhibiting TLR4/NF-кB/HIF-1α signaling pathway
Source: Front Physiol. 2025 Jul 1;16:1573405. doi: 10.3389/fphys.2025.1573405 (PMC12259664; doi:10.3389/fphys.2025.1573405)
Supplement: Supplementary file 5 [file Table3.docx]

Supplementary Table S3

The primer sequences

| TLR4 | F:CAAATATGCCTGTGCTGAGTTT | R:GACGCTTTCACCCCTACCA |
| --- | --- | --- |
| NF-κB-p65 | F:CTCTGTCGCCGTCTTCCA | R:GATAGGTCCTTCTTGCCCGTA |
| Galectin-3 | F:ACTGCCTGTGCCTTATGACC | R:TCTTGAAATCCAAAGCGAGTC |
| IL-6 | F:TAGCCCTCGTAGATGGGCA | R:CCCAGATCATGTTCGAGACGT |
| TNF-α | F:CCTTGTTCGGGTAGGAGACG | R:AGTAGCAAACCCGCAAGTGG |
| β-actin | F: CCTTGTTCGGGTAGGAGACG | R:AGTAGCAAACCCGCAAGTGG |
